# Inferring early genetic progression in cancers with unobtainable premalignant disease

**DOI:** 10.1038/s43018-023-00533-y

**Published:** 2023-04-20

**Authors:** Ignaty Leshchiner, Edmund A. Mroz, Justin Cha, Daniel Rosebrock, Oliver Spiro, Juliana Bonilla-Velez, William C. Faquin, Armida Lefranc-Torres, Derrick T. Lin, William A. Michaud, Gad Getz, James W. Rocco

**Affiliations:** 1 Broad Institute of MIT and Harvard, Cambridge, MA USA; 2 Department of Otolaryngology—Head and Neck Surgery, The Ohio State University Wexner Medical Center, Columbus, OH USA; 3 The James Cancer Hospital and Solove Research Institute, The Ohio State University, Columbus, OH USA; 4 Department of Otolaryngology—Head and Neck Surgery, Massachusetts Eye and Ear, Boston, MA USA; 5 Department of Pathology, Massachusetts Eye and Ear, Boston, MA USA; 6 Department of Pathology, Massachusetts General Hospital, Boston, MA USA; 7 Department of Surgery, Massachusetts General Hospital, Boston, MA USA; 8 Cancer Center, Massachusetts General Hospital, Boston, MA USA; 9 Harvard Medical School, Boston, MA USA; 10 The Ohio State University Comprehensive Cancer Center—James, The Ohio State University, Columbus, OH USA

**Keywords:** Cancer genomics, Head and neck cancer, Computational biology and bioinformatics, Cancer

## Abstract

Analysis of premalignant tissue has identified the typical order of somatic events leading to invasive tumors in several cancer types. For other cancers, premalignant tissue is unobtainable, leaving genetic progression unknown. Here, we demonstrate how to infer progression from exome sequencing of primary tumors. Our computational method, PhylogicNDT, recapitulated the previous experimentally determined genetic progression of human papillomavirus-negative (HPV^–^) head and neck squamous cell carcinoma (HNSCC). We then evaluated HPV^+^ HNSCC, which lacks premalignant tissue, and uncovered its previously unknown progression, identifying early drivers. We converted relative timing estimates of driver mutations and HPV integration to years before diagnosis based on a clock-like mutational signature. We associated the timing of transitions to aneuploidy with increased intratumor genetic heterogeneity and shorter overall survival. Our approach can establish previously unknown early genetic progression of cancers with unobtainable premalignant tissue, supporting development of experimental models and methods for early detection, interception and prognostication.

## Abstract (web summary)

Getz and colleagues demonstrate a computational approach to estimate the timing of genomic drivers in early tumor progression from cancer types that do not have premalignant disease, using HPV-negative and HPV-positive head and neck squamous cell carcinoma as examples.

## Main

Deciphering the temporal order of genetic lesions throughout the steps of cancer progression has long been a goal of cancer research^1,2^. That order can provide clues to etiology and cell-intrinsic mechanisms in tumorigenesis, informing studies of how normal tissue becomes a tumor, and can also provide ways to detect and treat early disease stages and identify early clonal events as promising targets for therapy of later invasive tumors^3^.

For cancers with well-defined pathologic progression from normal tissue, for example, adenoma through carcinoma in situ to invasive carcinoma, analysis of lesions along that trajectory has provided corresponding genetic progression models^4–13^. However, there are many cancer types with poorly defined, undetectable or difficult-to-biopsy premalignant lesions^14–22^ whose genetic progression thus remains speculative.

Here, we demonstrate how to infer the typical order of genetic events in a cancer type from exome sequencing of primary tumor samples taken long after cancer initiation. The genome of an invasive tumor includes information about its initial genetic progression, which is recorded in patterns of somatic mutations and copy number changes as cells evolve into an invasive clone^23,24^. We recently developed PhylogicNDT, an integrated suite of tools, to reconstruct the clonal architecture and relative timings of genetic events using a coherent probabilistic framework^25,26^ and have successfully applied it in several contexts^25,27–29^.

We asked whether more readily available whole-exome sequencing (WES) of primary tumors could similarly provide information on genetic progression.

**Fig. 1:**
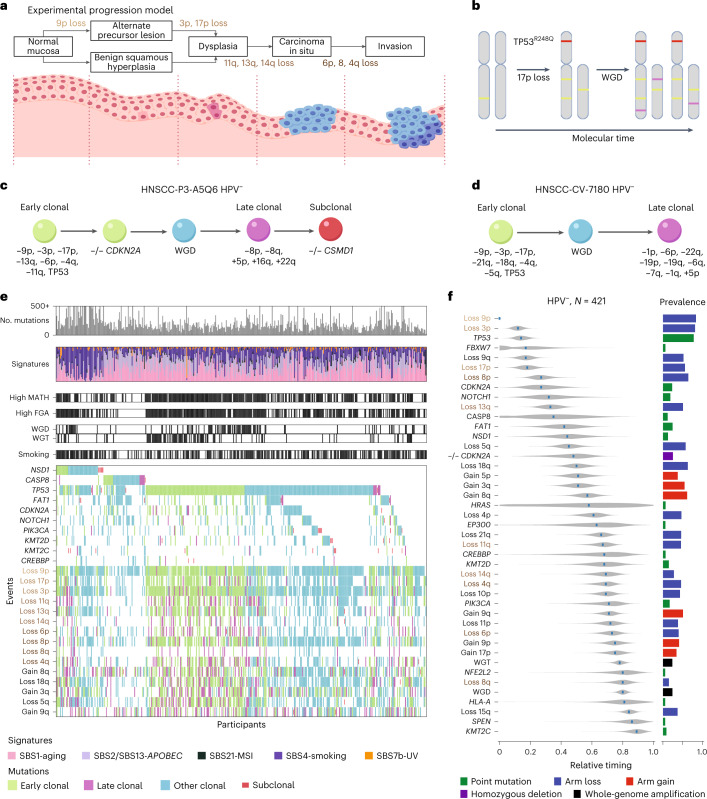
Genetic events and their timing in HPV^–^ HNSCC. **a**, Diagram of HPV^–^ genetic progression determined by Califano et al.^8^ from tissue samples taken near the tissue surface at different stages of disease progression with associated genetic events (left to right). **b**, Conceptual basis of estimating timing within tumors. The loss of chromosome arm 17p most likely occurred before the WGD event. The mutation *TP53*^R248Q^ most likely occurred before WGD, based on estimated multiplicity. Additional mutations (purple) are used to estimate the mutational relative timing of the WGD event. **c**,**d**, Examples of timing of genetic events in individual tumor samples; –, chromosome arm loss; +, chromosome arm gain; –/–, homozygous deletion in the indicated gene. **e**, CoMut plots for 421 HPV^–^ HNSCCs. For each tumor, from top to bottom, the number of mutations, their mutational signatures, MATH, FGA, the presence of whole-genome amplification (WGD and WGT), smoking within 15 years of diagnosis and selected genetic events are shown. The shading corresponds to timing of individual events. MSI, microsatellite instability. **f**, Relative timing of genetic events based on 421 HPV^–^ HNSCCs. The analysis compared 43 events among tumors: whole-genome amplification (WGD and WGT), arm losses examined by Califano et al.^8^ (names are colored with respect to timing groups in **a**) and other events with notable prevalence among HPV^–^ tumors (Methods). Events are ordered top to bottom by the point estimates of their mRT scores. Violin plots illustrate the posterior distributions of relative timing. The event prevalence and type (color coded) are displayed to the right of the corresponding violin plot. Source Data

We examined head and neck squamous cell carcinoma (HNSCC), which provides a unique opportunity to validate and extend such progression models. A quarter of a century ago, Califano et al.^8^ determined the genetic progression of classic HNSCC, typically associated with tobacco and alcohol use, from loss-of-heterozygosity (LOH) analysis along the pathologic progression from normal tissue to initial lesion, dysplasia and carcinoma in situ to invasive carcinoma (Fig. 1a). That model, as confirmed and extended to other genetic events in subsequent studies^30–34^, continues to provide the framework for HNSCC progression^35,36^. A valid computational reconstruction of genetic progression from WES of primary HNSCC should agree with those findings and provide timing for additional genetic events.

In contrast to classic HNSCC, the genetic progression of the increasingly prevalent and clinically important HNSCC associated with human papillomavirus (HPV;^37,38^ abbreviated HPV^+^ HNSCC) has remained unknown^39^. Premalignant lesions have not been identified reliably for HPV^+^ HNSCC^39,40^. The early genetic events in classic HPV^–^ HNSCC that disrupt the *CDKN2A* and *TP53* loci are infrequent in HPV^+^ HNSCC, as the E7 and E6 viral protein products provide corresponding tumorigenic functions^41,42^. Whether HPV^+^ HNSCCs converge thereafter to the same genetic progression as HPV^–^ HNSCC is unclear. Computational reconstruction provides a unique opportunity to determine the genetic progression of HPV^+^ HNSCC and explore whether the well-known differences between HPV^+^ and HPV^–^ HNSCC in response to therapy^35^ are associated with their different genetic paths to invasive cancer.

We thus applied PhylogicNDT to WES data from primary HNSCC to (1) demonstrate the reconstruction of genetic progression in HPV^–^ HNSCC and (2) identify the genetic progression of HPV^+^ HNSCC. We started with HNSCC from The Cancer Genome Atlas (TCGA) project^43^ and collected samples from 43 additional oropharyngeal HNSCCs to increase the representation of HPV^+^ cases (101 total HPV^+^ HNSCCs).

Here, we report the validation of our computational approach in HPV^–^ HNSCC, extending the number and details of timed events, and describe the previously unknown order of genetic progression of HPV^+^ HNSCC. We document the timing of different types of aneuploidy and their strong association with intratumor heterogeneity and survival. Additionally, we converted the relative timing estimates of main drivers and HPV integration to years before diagnosis based on a clock-like mutational signature.

This report provides a framework for studying genetic progression of cancer types for which premalignant tissue is unobtainable. This framework enables uncovering mechanisms of cancer initiation and early development in types of cancer for which the rarity, inaccessibility, or lack of premalignant tissue previously made their genetic progressions undecipherable.

## Results

The data in Fig. 1b show how the order of genetic events during tumor development is inferred from a tumor sample obtained long after initiation, once the fraction of cancer cells that harbor each mutation (cancer cell fractions (CCFs)) and their multiplicities (the number of DNA molecules carrying a mutation per cancer cell) are determined. In this example, cancer cells in the final tumor sample (Fig. 1b, right) showed four copies of chromosome arm 17q but only two copies of arm 17p, consistent with LOH at 17p followed by duplication. The final pattern of somatic mutations (colored bands) was consistent with some mutations (green) before the duplication and others (purple) thereafter. The driver mutation in *TP53* (red) leading to a R248Q protein alteration was seen in both final copies of arm 17p, consistent with the mutation preceding the duplication.

Relative timing of all genetic events within each tumor is estimated across the genome by applying these principles in a probabilistic computational framework^25,26^. Examples of inferred orders of events in two HPV^–^ tumors are shown in Fig. 1c,d. The inferred timing of each event is represented as a posterior distribution over a molecular mutational time scale *π*, with *π* = 0 and *π* = 1 reflecting the times of the first and last clonal events, respectively^26^.

Next, PhylogicNDT combines the within-tumor timing (*π* value distributions) of selected driver events across a cohort of tumors to obtain a posterior distribution for the typical relative timings of the events. The median relative timing (mRT) for each genetic event provides a point estimate for ordering events into a genetic progression model.

We applied PhylogicNDT to WES data of 531 HNSCC tumor–normal pairs, with 421 HPV^–^ and 101 HPV^+^ tumors. We focused on 64 established or candidate HNSCC drivers, including 24 frequently mutated genes and 40 frequently gained or lost genomic regions in either or both HPV^–^ and HPV^+^ tumors (Supplementary Tables 1 and 2).

### Corroborating timing of driver events in HPV^–^ HNSCC

The genomic landscape of 421 HPV^–^ tumors is illustrated in Fig. 1e. For each participant’s tumor, we report the number of mutations, relative activity of different mutational signatures, measures of genetic heterogeneity, smoking history and the occurrence of the most frequent 10 driver genes and 15 copy number alterations, with tumor-specific timing estimates.

We computationally inferred the order of events among these HPV^–^ HNSCCs and compared the order to the empirically derived progression model of Califano et al.^8^ (Fig. 1a). In that model, preneoplastic tissue arises by loss of chromosome arm 9p (−9p; where *CDKN2A* resides), progressing to dysplasia with −17p (which includes *TP53*) and −3p. The transition to carcinoma in situ is associated with −13q, −11q and −14q, and, finally, progression to invasive cancer is associated with −6p, −8 and −4q. Even the partial orderings of within-tumor timing estimates were generally consistent with the Califano model (Fig. 1b–e). We combined the participant-specific orders of events across the cohort (using the PhylogicNDT LeagueModel tool; Methods) to obtain the typical order of the 43 most prevalent driver events (Fig. 1f).

Computational timing analysis, based solely on primary tumor specimens, recapitulated and substantially expanded the empirical progression model of Califano et al. (Fig. 1a). Notably, losses described by Califano et al. appeared in our model in the same order as in their model. We predicted that timings of losses of the p and q arms of chromosome 8 are different (*P* = 6.2 × 10^−5^). Indeed, the early loss of arm 8p, not evaluated by Califano et at., agreed with its frequent loss in early oral cavity dysplasia^31^.

The first and most prevalent event was –9p (prevalence of 84.6%; mRT = 0.05), the location of the *CDKN2*A tumor suppressor that is also inactivated by frequent (23.5%) and early (mRT = 0.27) point mutations or homozygous deletions (25.4% prevalence; mRT = 0.48). Nearly all HPV^–^ HNSCC (388/421; 92.2%) had at least one such disruption of *CDKN2A*.

Disruption of *TP53* was also frequent and early via mutation (78.4%; mRT = 0.14) or −17p (54.4%; mRT = 0.18), with at least one such event in 369 HPV^–^ tumors (87.6%). Our timing of *TP53* and *CDKN2A* single-nucleotide variations (SNVs) agreed with the long-appreciated presence of *TP53* (ref. ^30^) and *CDKN2A* (ref. ^32^) mutations in dysplastic tissue of the head and neck. The third of the earliest genetic events identified by Califano et al., −3p, occurred in 339 tumors (80.5%); its mRT of 0.12 was similar to that of *TP53* disruptions.

We further identified prevalent and early −9q (51.5%; mRT = 0.17) and −8p (62.9%; mRT = 0.27) events. By contrast, the frequent +3q event (55.6%), containing the *PIK3CA* locus, was later (mRT = 0.51), suggesting a role in progression rather than initiation. Other arm-level gains and losses covered a wide range of relative timings.

Based on the Califano et al. model, we mapped relative timings to pathologic progressions. Precursor lesions become dysplastic near an mRT of ~0.25, between timings of −17p (0.18) and −13q (0.33). The transition to carcinoma in situ is near 0.7 (mRT of −14q and −4q) and that to invasive carcinoma at or after ~0.75 (−6p mRT = 0.73; −8q mRT = 0.8). Our timing of +3q (mRT = 0.51) and +8q (mRT = 0.57) before this estimate of the transition to carcinoma in situ agrees with findings (published after the Califano model) of these gains in dysplastic tissue^33,34^.

We further established relative timing of SNVs in several lower-prevalence driver genes (in order of increasing mRT): *FBXW7*, *NOTCH1*, *CASP8*, *FAT1*, *NSD1*, *HRAS*, *EP300*, *CREBBP*, *KMT2D*, *PIK3CA*, *NFE2L2*, *HLA-A*, *SPEN* and *KMT2C*. mRT values of events in the first nine genes were less than 0.69, supporting early roles in tumorigenesis before carcinoma in situ. Although individually of low prevalence (each <25%), a mutation in at least one of these first nine genes occurred in over 60% of HPV^–^ HNSCCs (260/421). Mutations in *PIK3CA*, *NFE2L2*, *HLA-A*, *SPEN* and *KMT2C* were at later mRT values, presumably late in pathologic progression.

Finally, whole-genome events, leading to whole-genome copy number profiles that are predominantly triploid (WGT) or whole-genome doubling of both alleles (WGD) resulting in tetraploid samples, occurred in nearly half (47.7%) of HPV^–^ HNSCCs. These whole-genome events were late in progression (mRT values of 0.78 (WGT) and 0.80 (WGD); Fig. 1f).

### Establishing the genetic progression of HPV^+^ HNSCC

**Fig. 2:**
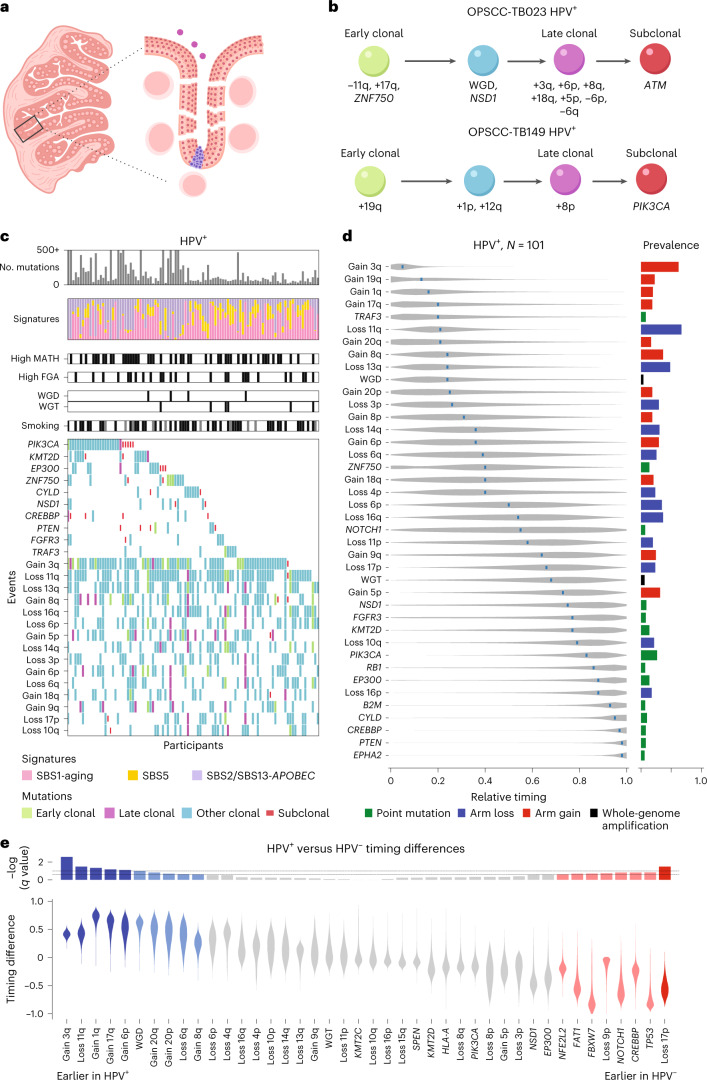
Genetic events and their timing in HPV^+^ HNSCC. **a**, Illustration of lack of sampling access and ease of tumor invasion with HPV^+^ tumors. Left, sketch of tonsil cross-section showing crypts; right, magnification of the base of a crypt illustrating entry of HPV (purple circles) and subsequent tumor development (blue) at a break in the discontinuous basement membrane. **b**, Examples of genetic event timing within individual tumor samples, represented as in Fig. 1c; OPSCC, oropharyngeal squamous cell carcinoma*.* **c**, CoMut plots for HPV^+^ HNSCC, represented as in Fig. 1e. The shading corresponds to timing from individual times with PhylogicNDT. The top 10 SNVs and 15 copy number variations by prevalence were selected for display. **d**, Relative timing of genetic events based on 101 HPV^+^ HNSCCs, represented as in Fig. 1f. The analysis compared 40 events among HPV^+^ tumors: whole-genome amplification (WGD and WGT), arm losses with >15% prevalence and SNVs with >5% prevalence. **e**, Relative timing between HPV classes of 42 shared events. Violin plots show the distributions of difference in HPV-class-specific timing. *P* values were derived from the MCMC posterior, as described in the Methods. Associated log_10_ (*q*) values are displayed at the top; *N* = 101 individuals with HPV^+^ HNSCC (**b**–**d**). Source data

Encouraged by our success with classic HPV^–^ HNSCC, we turned to HPV^+^ HNSCC, where pathologic progression has not been identified^35,44^. These tumors typically arise in crypts of tonsils and related lymphoid tissues at breaks of the basement membrane^35,45,46^ that allow viral access to infect basal epithelial cells and ready entry of transformed cells into the lymphatics (Fig. 2a). HPV^+^ HNSCC often spreads to local lymph nodes before a primary tumor is identified; premalignant tissue has seldom been found^35,47^.

**Extended Data Fig. 1:**
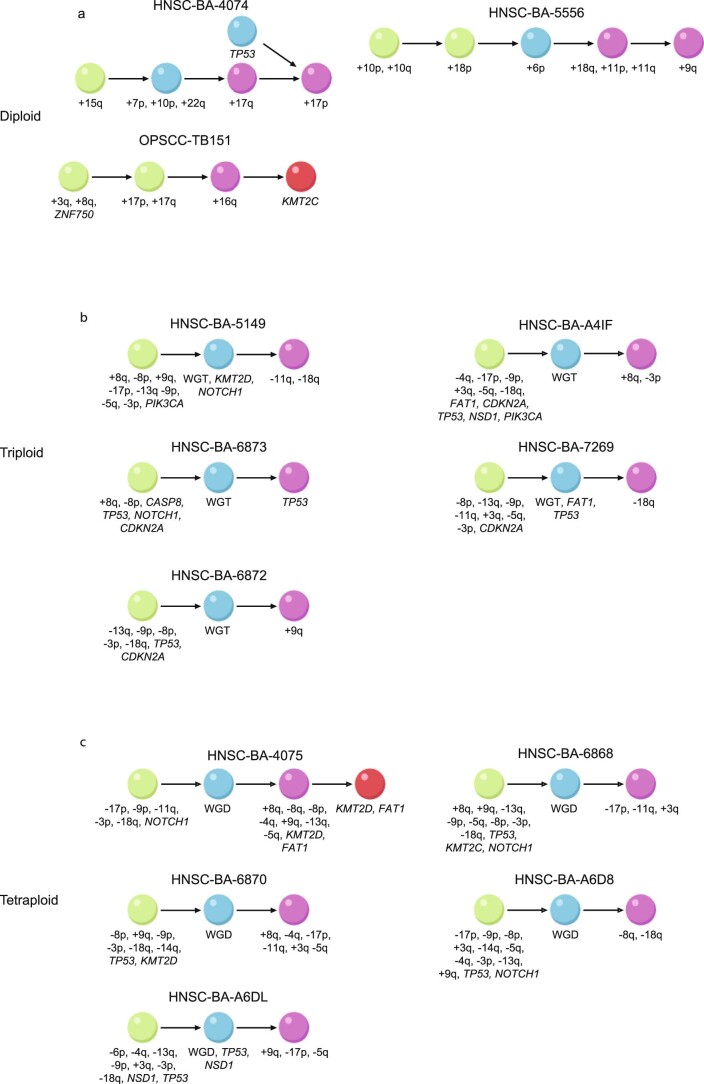
Single-patient timing. Examples of genetic event timing within individual tumor samples classified as diploid **(a)**, triploid **(b)**, and tetraploid **(c)**. WGT, whole-genome triploidy; WGD, whole-genome duplication; –, chromosome arm loss; +, arm gain; other symbols, mutation in the indicated gene. The colors of the balls represent the timing. Green, early clonal; purple, late clonal; blue, other clonal; red, subclonal.

We assembled WES data from 101 HPV^+^ HNSCCs, including 65 from TCGA^43^ and 36 collected for this study. Examples of within-tumor timing are shown in Fig. 2b and Extended Data Fig. 1; the genetic landscape of HPV^+^ HNSCCs is illustrated in Fig. 2c. We found four mutational signatures in this cohort, with 28% of mutations attributed to aging signatures (single-base substitution 1 (SBS1) and SBS5) and 62% to *APOBEC* signatures (SBS2 and SBS13). Median tumor purity was 0.48 (range of 0.12 to 0.98), and median ploidy was 2.14 (range of 1.60 to 5.56; Methods).

As for HPV^–^ HNSCC, we estimated the order of events (*π*) for each HPV^+^ tumor via PhylogicNDT SinglePatientTiming (Fig. 2c). We then combined timing information for 40 genetic driver events across tumors to infer the typical order in HPV^+^ cancers (Fig. 2d).

Gain of chromosome arm 3q and loss of 11q were both highly prevalent and early (+3q: 70.3% prevalence and mRT = 0.05; −11q: 69.3% prevalence and mRT = 0.20). Arm-level events −13q and +8q were also both highly prevalent (>40%) and early (mRT values of 0.21 and 0.24, respectively), suggesting frequent roles in early HPV^+^ progression. All other arm-level events had a prevalence of <40%.

Of 13 low-prevalence potential driver genes, mutations only in *TRAF3*, *ZNF750* and *NOTCH1* had mRT values of 0.55 or earlier, while others had mRT values of 0.75 or greater. Except for *PIK3CA* (mRT = 0.83), none of the 13 potential driver genes were mutated in more than 15% of HPV^+^ tumors, although nearly three-quarters of tumors (74/101, 73%) had a mutation in at least 1 potential driver gene. Only 10 of 101 HPV^+^ HNSCCs had genome-wide events leading to WGT or WGD.

### Comparing HPV^+^ and HPV^–^ genetic progressions

In HPV^+^ HNSCC, the HPV E7 and E6 viral gene products inactivate the functions of *CDKN2A* and *TP53*, whereas in HPV^–^ HNSCC, those functions are lost via somatic events^41,42^. We sought to determine other similarities and differences in the progression of these two major HNSCC subtypes.

First, we compared the frequencies of genomic events in HPV^+^ and HPV^–^ HNSCC. Even beyond expected differences associated with *CDKN2A* and *TP53*, 20 genetic events were significantly more prevalent in HPV^–^ HNSCC, including SNVs in *FAT1*, *NOTCH1*, *CASP8*, *HRAS*, the −3p highly prevalent in HPV^–^ HNSCC and 15 other arm-level somatic copy number alterations (Supplementary Tables 1 and 2; Fisher’s exact test with Benjamini–Hochberg false discovery rate *q* value of <0.1). Notably, whole-genome events leading to WGT or WGD were nearly five times more prevalent in HPV^–^ (201/421, 48%) than in HPV^+^ tumors (10/101, 10%; *P* = 10^−13^, Fisher’s exact test). However, 14 events were found at higher prevalence in HPV^+^ than in HPV^–^ HNSCC (SNVs in *PIK3CA*, *ZNF750*, *EP300*, *CYLD*, *TRAF3*, *FGFR3*, *PTEN*, *B2M* and *RB1* and arm-level events +3q, −11q, −16q, −18q and +19q).

Comparing mutational signatures showed that the prevalence of all signatures differed between HPV^+^ and HPV^–^ tumors, with *APOBEC* (SBS2/SBS13) and aging signatures (SBS1/SBS5) enriched in HPV^+^ tumors and the smoking signature (SBS4) enriched in HPV^–^ tumors.

Next, to compare the timing of events, we examined 42 events present in at least three cases in each of the HPV^+^ and HPV^–^ cohorts (Fig. 2e). We combined all anatomic sites, as the few (33) HPV^–^ oropharyngeal tumors did not allow reliable anatomic site-specific timing comparisons. We calculated distributions of differences in relative timing and assessed statistical significance by permutation (Methods). We detected 6 differentially timed events at *q* < 0.1 (5 earlier in HPV^+^ and 1 in HPV^–^) and 12 more at *q* < 0.2.

Early high-prevalence genetic events in HPV^–^ progression, −9p, −17p and mutation of *TP53* (*CDKN2A* or *TP53* inactivation), were earlier than in HPV^+^ tumors, as expected from the different mechanisms for inactivating the function of these tumor suppressor genes. Lower-prevalence mutations in *CREBBP*, *NOTCH1*, *FBXW7*, *FAT1* and *NFE2L2* were also earlier in HPV^–^ tumors.

The early and high-prevalence genetic events in HPV^+^ progression, +3q and −11q, were significantly earlier in HPV^+^ than in HPV^–^ progression, supporting their roles as early HPV^+^ driver events. WGD in HPV^+^ HNSCC was also found to occur significantly earlier than in HPV^–^ tumors, despite the much lower prevalence of WGD in HPV^+^ tumors. Other arm-level events earlier in HPV^+^ progression were gains +1q, +17q, +6p, +20p, +20q and +8q and loss of 6q.

### Progressions in HNSCC subclasses and at anatomic subsites

**Fig. 3:**
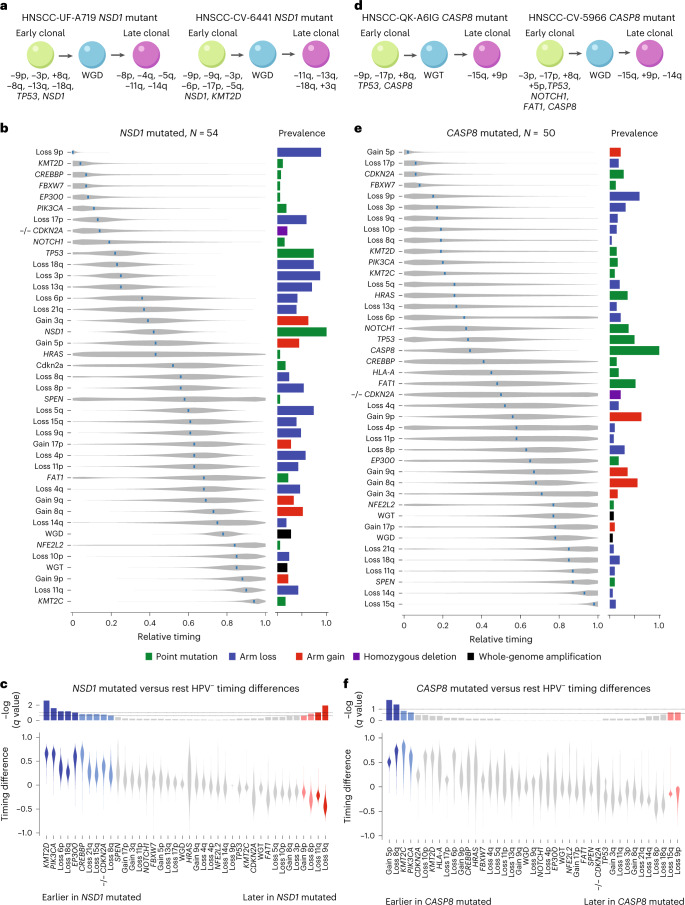
Timing within subsets of HPV^–^ HNSCC. **a**, Examples of single-tumor timing diagrams for tumors with mutations in *NSD1*. **b**, Relative timing diagram for 54 HPV^–^ tumors with mutations in *NSD1*, represented as in Figs. 1f and 2d. **c**, Comparison of event timing between the *NSD1*-mutated and the remaining HPV^–^ HNSCCs, represented as in Fig. 2e. **d**–**f**, Corresponding example of single-tumor timing diagram (**d**), relative timing diagram (**e**) and comparison of event timing diagram (**f**) for 50 tumors with mutations in *CASP8*. Source data

**Extended Data Fig. 2:**
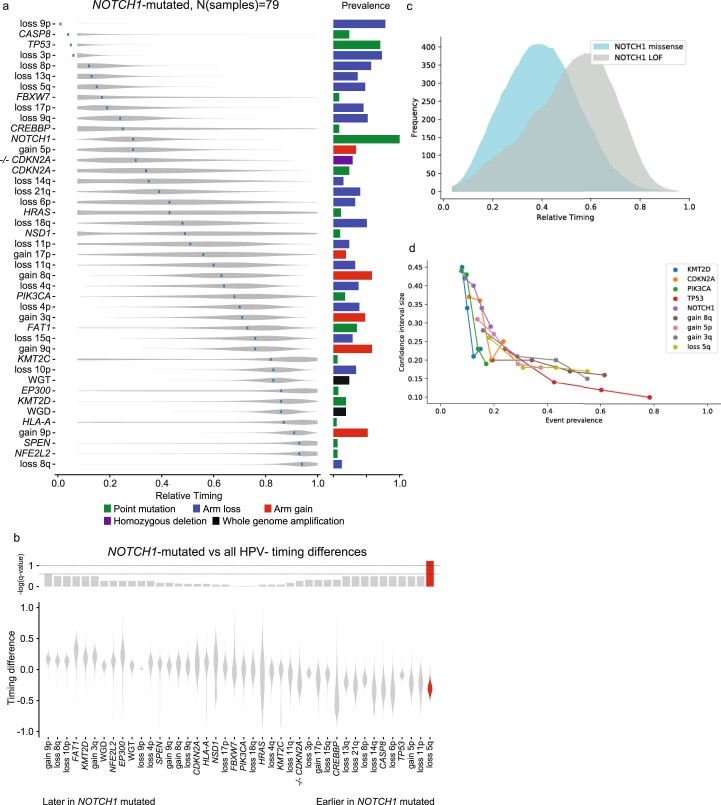
Timing within the *NOTCH1*-mutated subset of HPV- HNSCC. **(a)** Relative Timing of genetic events, based on 79 NOTCH1-mutated HPV-negative HNSCCs. Analysis compared 43 events among tumors: whole-genome amplification (WGD, doubling; WGT, triploidy) and other events with notable prevalence among HPV-negative tumors (arm losses with > 30% prevalence, SNV with > 5% prevalence). **(b)** Relative timing between NOTCH1-mutated and all HPV-negative tumors of 42 events (all events from panel a excluding NOTCH1) (N = 79 NOTCH1-mutated patients). Violin plots show the distributions of difference in timing between the groups. Associated log10 q-values are displayed at the top. **(c)** MRT timing profiles for NOTCH1 activating and deactivating mutations **(d)** Power analysis through down sampling of higher prevalence events, demonstrating dependence of the confidence interval size on event prevalence (N = 421 HPV- patients). Source data

Among HPV^–^ HNSCC, prevalence of some genetic events differed markedly among the major anatomic subdivisions oral cavity, oropharynx and larynx. *NSD1* mutations were preferentially laryngeal, while almost all *CASP8* mutations were in oral cavity tumors (Supplementary Table 3). That led to the question of whether tumors with these mutations, reflecting distinct anatomic sites, have different genetic progression trajectories. We used PhylogicNDT to infer genetic progression of HPV^–^ HNSCC subsets with mutations in *NSD1* (*n* = 54) or *CASP8* (*n* = 50), comparing their timings against other HPV^–^ tumors (Fig. 3). We also estimated timing in *NOTCH1*-mutated tumors (*n* = 54), which showed no significant subsite preference (Extended Data Fig. 2a–c).

The preferentially laryngeal *NSD1*-mutated tumors (Fig. 3a–c) had several events whose timing differed from other HPV^–^ HNSCCs, despite typically similar event prevalence between those two groups (Supplementary Table 4). Mutations in *KMT2D*, *CREBBP*, *EP300* and *PIK3CA* timed at an mRT of ≤0.11 in *NSD1*-mutated cases versus at an mRT of ≥0.63 in other HPV^–^ HNSCCs. Homozygous deletions of *CDKN2A* were also significantly earlier in *NSD1*-mutated tumors. *NSD1* mutations themselves had an mRT of 0.42. Significantly later events were arm-level losses or gains. Some timing differences could be associated with increased smoking signature (SBS4) activity in *NSD1*-mutated tumors (Fig. 1b).

By contrast, *CASP8*-mutated tumors, almost solely in the oral cavity, showed major differences in event prevalence from other HPV^–^ HNSCCs (Supplementary Table 5), while mRT values of most events were similar (Fig. 3e–f). Compared to other HPV^–^ HNSCCs, they had a substantially lower prevalence of whole-genome events (14% versus 52.3%; *P* = 10^−7^, Fisher’s exact test). Nearly two-thirds of driver events (46 of 65) had differential prevalence (*q* < 0.1; Supplementary Table 4). Events more frequent in *CASP8*-mutated tumors included +9p (64% versus 39.1%) and mutations in *HRAS* (36% versus 2.7%), *FAT1* (52% versus 20.8%), *EPHA2* (18% versus 2.4%), *EP300* (18% versus 4.6%) and *NOTCH1* (38% versus16.2%). *TP53* (50% versus 82.2%) and *NSD1* (4% versus 14%) mutations and losses −17p (18% versus 59.3%), −3p (30% versus 87.3%) and −11q (8% versus 46.4%) were significantly less prevalent. *CASP8*-mutated tumors thus are evidently driven more by mutations in multiple driver genes than by chromosome arm gains or losses. Single-individual timing diagrams of *CASP8*-mutated cases show early *CASP8*, *TP53* and *CDKN2A* mutations (Fig. 3d). *CASP8* mutation itself had an mRT of 0.34 (Fig. 3e), similar to that of *NSD1* in the *NSD1*-mutated subset. Four events were significantly earlier in *CASP8*-mutated HPV^–^ HNSCC than in other HPV^–^ HNSCC (Fig. 3f), although of these, only +5p had a prevalence higher than 20%. Two losses, −9p and −15q, were significantly later in *CASP8*-mutated tumors.

*NOTCH1*-mutated tumors showed only few differences from other HPV^–^ HNSCCs in event prevalence or timing, including a higher prevalence of mutations in *CASP8* (24.1% versus 9.1%) and lower prevalence of +17p (19.0% versus 31.0%) and +8p (3.8% versus 17.3%; Supplementary Table 6). Only −5q showed different timing from other HPV^–^ HNSCCs (mRT of 0.15 versus 0.45; Extended Data Fig. 2), although fewer events and individuals in this subtype might have limited the power of this analysis (Extended Data Fig. 2d).

**Extended Data Fig. 3:**
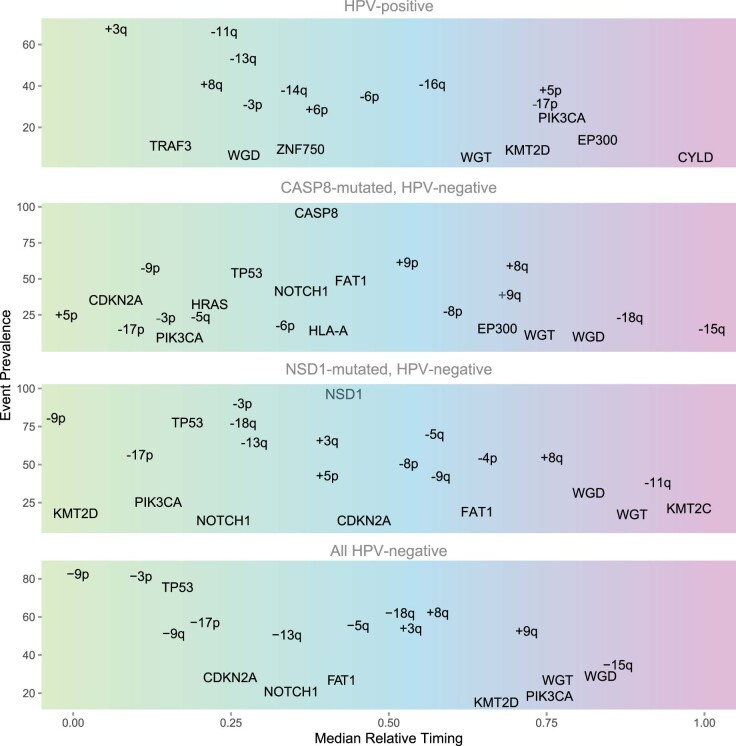
Summary of the developmental trajectories of the main subtypes of HNSCC. Prevalence versus median Relative Timing (mRT) estimates for major events in subsets of HNSCC, from top to bottom: HPV+, CASP8-mutated HPV–, NSD1-mutated HPV–, all HPV–.

Overall, timing analysis of distinct tumor subtypes can detect differential ordering of events independent of prevalence and associated subtype-specific orders with the unique biology of each subtype (Extended Data Fig. 3).

### Genetic heterogeneity, aneuploidy and progression

**Fig. 4:**
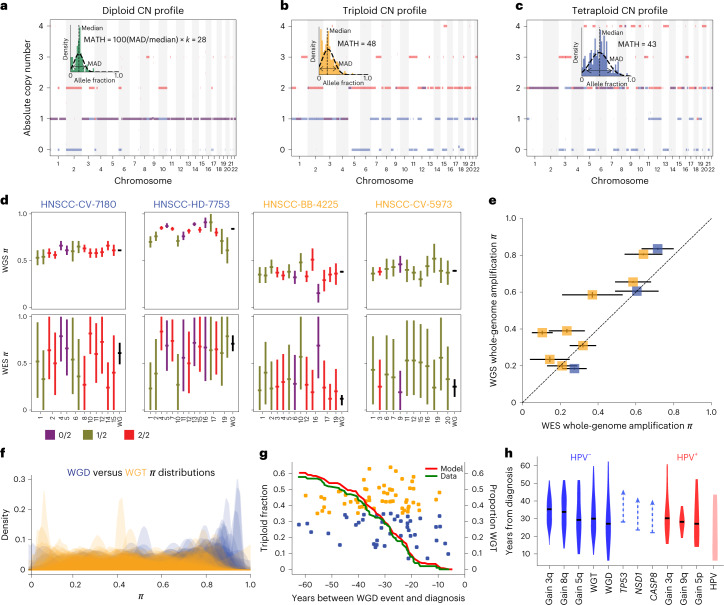
Whole-genome events and aneuploidy classes in HNSCC. **a**–**c**, Allelic copy number (CN) profiles after ABSOLUTE in example tumors with diploid (**a**), triploid (**b**) and tetraploid (**c**) profiles. Each locus has a blue line and a red line representing the copy numbers of the minor and major alleles, respectively. Histograms of raw MAF distributions among somatically mutated loci and corresponding MATH values (scale factor of *k* = 1.4826 for the median absolute deviation (MAD) of a normal distribution to equal its standard deviation) are shown. **d**, Comparison of whole-genome amplification timing determined from WES against that from WGS of the same tumors (*N* = 4). Each chromosome arm is timed individually and aggregated to determine the timing of the whole-genome event. Left, two tumors with a tetraploid profile. Right, two tumors with a triploid profile. Horizontal ticks represent means. Error bars represent 75% credible intervals from posterior sampling. Colors represent copy number states. **e**, Scatter plot of whole-genome amplification timings in WGS versus WES of 11 tumors. Boxes represent timing means. Error bars represent 75% credible intervals from posterior sampling; blue, tetraploid profile; yellow, triploid profile. **f**, Timing probability distributions across tumors (*N* = 216 whole-genome amplifications), on *π* scale, of whole-genome amplifications leading to tetraploid (WGD) and triploid (WGT) copy number profiles. **g**, Real-time timing of WGT and WGD events. Estimated conversion rate of WGD events into WGT per year with a Poisson-like model (red model and green data; *N* = 103 real-timed whole-genome amplifications). **h**, Real-time timing of main driver events in HPV^+^ (red) and HPV^–^ (blue) tumors (*N* = 205 individuals with real-timed driver events). Arrows for somatic point mutations represent that the estimate is late bound by the time of the regional gain, but no early bound exists. HPV integration sites include early events and events occurring during the development of the tumor. Source data

**Extended Data Fig. 4:**
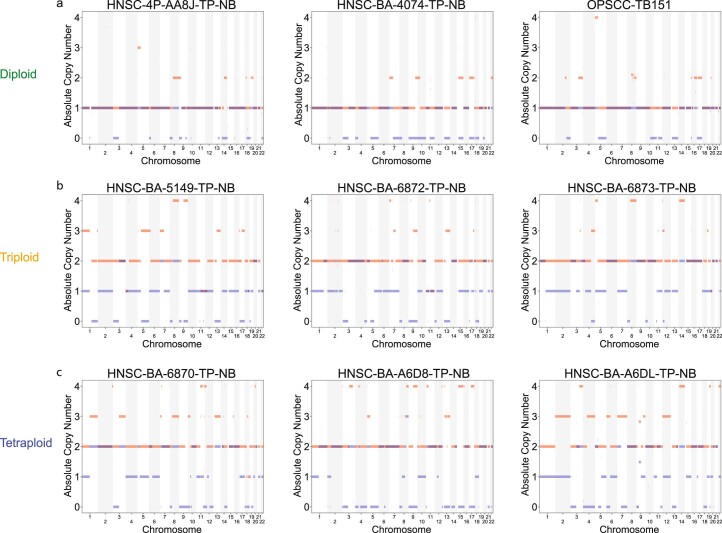
Absolute copy number profiles. Copy-number (CN) profiles from SNP arrays or whole-exome sequencing (WES; after ABSOLUTE correction for purity) of alleles in example tumors with diploid **(a)**, triploid **(b)**, and tetraploid **(c)** profiles. Each locus has a blue line and a red line representing the copy number of the minor and major alleles respectively. Source data

High levels of genetic heterogeneity in a tumor are associated with worse outcomes in HNSCC^48–51^ and other types of cancer^52^. In HNSCC, studies have used the mutant allele tumor heterogeneity (MATH) score as a measure of genetic heterogeneity, defined as the median-normalized width of the distribution of mutant allele fractions (MAFs; Fig. 4a–c and Extended Data Fig. 4)^48^. Heterogeneity of MAF values could arise from mutation multiplicity differences within cells or differences in mutations among subclones. PhylogicNDT analysis allowed us to investigate relationships between MATH and other measures of genetic heterogeneity, evaluate the genomic source of high MATH scores and estimate how the MATH score changes during progression.

We identified three classes of tumor aneuploidy based on absolute copy number profiles (Fig. 4). About half of tumors (Fig. 4a) showed essentially diploid profiles, with some LOH or copy number gains. Tumors with more disrupted copy number profiles, suggesting a genome-wide amplification during tumorigenesis, unexpectedly manifested as two distinct subtypes with similar prevalence: (1) triploid cancers with multiple genomic regions showing three total copies (WGT; Fig. 4b) and (2) tetraploid cancers with multiple genomic regions at four copies (two copies of each allele; Fig. 4c), suggesting a WGD event. Examples of single-tumor timing of these aneuploidy classes are in Extended Data Fig. 1.

**Fig. 5:**
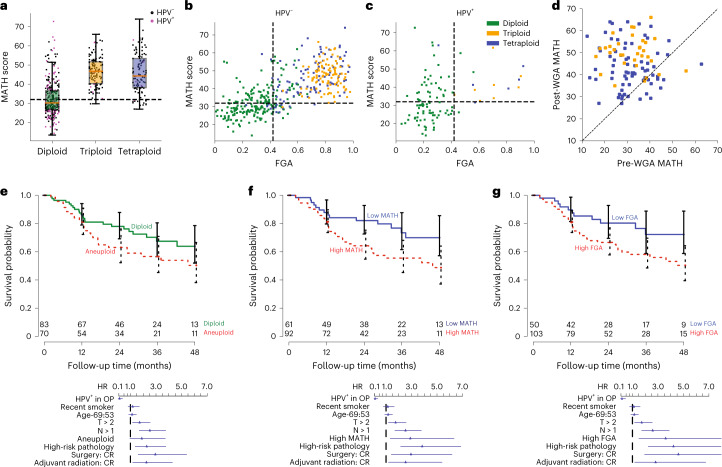
Relationships among measures of intratumor genetic heterogeneity and their associations with outcome. **a**, Box plots of MATH scores versus aneuploidy class for *N* = 522 total participants; red lines indicate the median, whiskers extend to the extreme values within 1.5× IQR of the box delimiting the first and third quartiles. **b**,**c**, Individual tumor MATH scores versus the FGA. Colors represent tumor aneuploidy classes. A Student’s *t*-test for correlation (bivariate data assumed but not tested) was conducted to obtain *P* values. Data are displayed for HPV^–^ (**b**) and HPV^+^ (**c**) tumors. **d**, Timing relationship between MATH and WGD. For tumors with timing of WGD, MATH calculated from post-WGD SNV (vertical axis) is plotted against that calculated from pre-WGD SNV (horizontal axis). **e**–**g**, Associations of intratumor heterogeneity measures with outcome. Top, Kaplan–Meier survival curves (with 95% log survival CIs) stratified by a heterogeneity measure for the participant subset with survival most associated with intratumor heterogeneity (surgery without adjuvant therapy or therapy involving chemoradiation; excluding HPV^+^ oropharyngeal tumors to avoid confounding with HPV; *N* = 153 participants). Numbers at risk are shown over time. Bottom, HR point estimates (with 95% Wald CI) from Cox multiple regression model on 441 participants. The model was stratified by anatomic site and included interactions of the heterogeneity measure with therapy received and with high-risk pathology (evidence of close or positive surgical margins or extranodal tumor extension). HRs are displayed for HPV^+^ oropharyngeal tumors versus others (HPV^+^ in OP), smoked within 15 years of diagnosis (recent smoker), 75th and 25th percentiles of age (Age-69:53), T classification greater than 2 (T > 2), N classification greater than 1 (N > 1), presence of high-risk pathology as defined above (high-risk pathology) and surgery without adjuvant radiation and surgery with adjuvant radiation alone versus those receiving chemoradiotherapy (CR) as primary therapy or adjuvant to surgery (Surgery: CR and Adjuvant radiation: CR), with aneuploidy as the heterogeneity measure (**e**), with high MATH (MATH > 32.7) as the heterogeneity measure (**f**) and with FGA as the heterogeneity measure (**g**); high FGA cutoff is at the same percentile among tumors as for MATH. Source data

**Extended Data Fig. 5:**
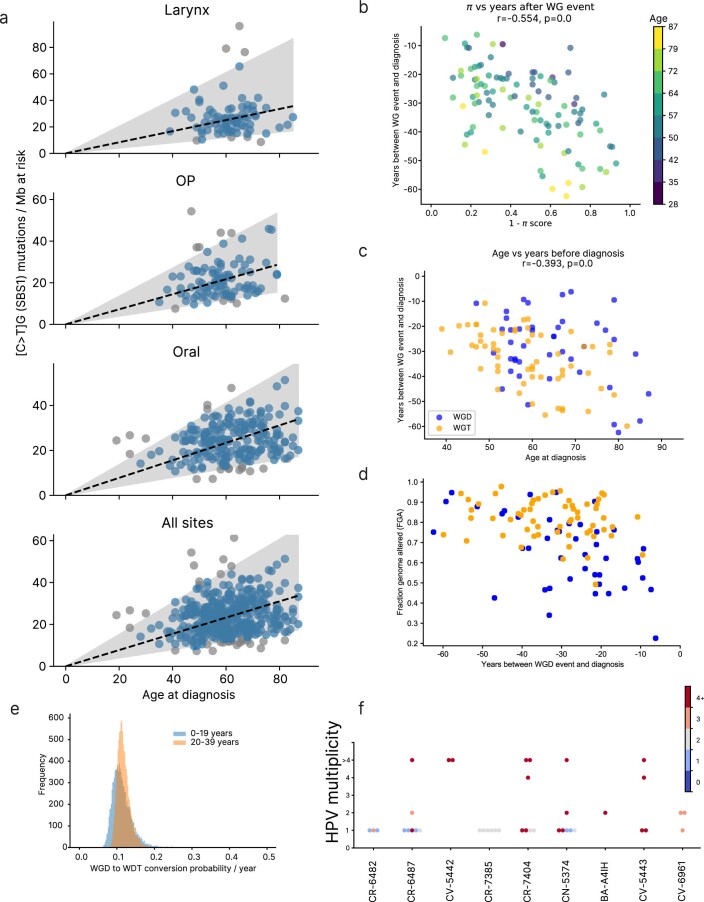
Realtime timing. **(a)** CpG mutation rate per bases at risk vs age of the patient. Robust linear regression with top and bottom 5% excluded (shaded area). (N = 413 patients) (b-d) Timing of WGT and WGD events in real time (b-e: N = 103 real-timed whole genome amplifications) **(e)** The posterior for Lambda in the Poisson process representing the conversion of WGD to WGT per year **(f)** HPV integration sites’ multiplicities for WGS data used in timing analysis (N = 52 HPV integration sites). Source data

We investigated timing differences between WGT and WGD events. WGT occurred significantly earlier in both WES and whole-genome sequencing (WGS) single-tumor data (rank-sum test, *P* < 0.001; Fig. 4d–f), suggesting that primary tumors with WGT profiles experienced early WGD events, providing sufficient time to delete genomic regions that brings the average copy number down to approximately three. More recent genome duplication in tetraploid tumors (labeled WGD) kept the average copy number close to four. Increased aneuploidy going from diploid to WGD and WGT stages (Fig. 4a–c and Extended Data Figs. 4 and 5) and wider distributions of MAFs (higher MATH scores) with higher aneuploidy support this interpretation. Higher aneuploidy classes were associated with higher MATH values (Fig. 5a) and were thus related to that clinically relevant measure^48–50^. The fraction of the genome altered (FGA), reflecting the genomic regions with copy numbers different from the modal copy number level, provided an overall measure of aneuploidy. Noticeably, this measure was near linearly related to the MATH score (Fig. 5b) and strongly associated with aneuploidy class. The distribution of aneuploidy classes, FGA and MATH scores differed significantly between HPV^+^ and HPV^–^ HNSCCs (Fig. 5b,c). The long-established relationship between HPV^+^ tumors and low MATH values^48^ is thus explained by lower prevalence of whole-genome events in HPV^+^ tumors.

As PhylogicNDT provided timing information for whole-genome events in single tumors, we could estimate MATH values before such events by only including mutations timed before a tumor’s whole-genome event (Fig. 5d). MATH values were generally much higher after WGD, presumably due to marked aneuploidy after doubling. Although high MATH is found in many diploid tumors (Fig. 5b,c), it is almost universal (213/233, 91.4%) in tumors with a whole-genome event.

**Extended Data Fig. 6:**
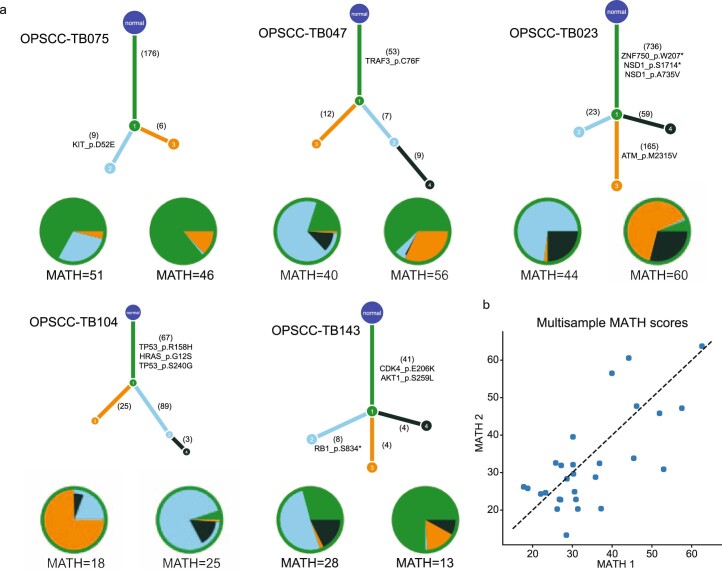
Multi-sample patient phylogeny and heterogeneity. **(a)** Examples of phylogenetic trees generated by PhylogicNDT (BuildTree module) for selected patients with two samples of the same tumor. Numbers on branches represent the number of SNVs or indels that are added along each branch. Pie graphs below represent cancer cell populations of each subclone, constrained by the pigeonhole principle, estimated by PhylogicNDT (BuildTree module). **(b)** Scatter plot of MATH scores from patients with multiple tumor samples. Each axis represents the MATH score from one of the samples. (N = 28 patients with multiple samples). Source data

Paired samples (two from the same tumor) were available for 28 oropharyngeal tumors newly collected for this study. Although the mutations and subclonal composition could differ substantially between paired samples (Extended Data Fig. 6a), their overall MATH and FGA measures of heterogeneity were similar (Extended Data Fig. 6b). These measures seem to be intrinsic characteristics of a tumor as a whole, supporting their clinical potential as biomarkers.

### Time between somatic events, including HPV integration, and diagnosis

We converted relative timing estimates to the expected number of years before diagnosis. We modeled CpG>T (‘aging’ signature SBS1) mutations (total number of mutations/number of covered CpG sites) as a function of participant age at diagnosis (Extended Data Fig. 5a). We observed an accumulation of 0.37 (interquartile range (IQR) of 0.29–0.51) CpG>T mutations per megabase at risk per year in HNSCC HPV^–^ tumors and a similar value of 0.39 (IQR of 0.25–0.45) in HPV^+^ tumors. We found that these rates were also similar among tumor anatomical sites (Extended Data Fig. 5a). We then used these rates to convert participant-specific CpG>T *π* estimates for driver events to real time, measured in years before diagnosis (Extended Data Fig. 5a–d).

Real-time timing estimates of WGT and WGD support a model with an abrupt transition from the WGD state to WGT (Fig. 4g, Extended Data Fig. 5c–e and Supplementary Table 7). Only two triploid tumors had the WGD event estimated to occur during the 15-year period before diagnosis, whereas seven WGD cases did not transition to WGT in that time frame (Fisher’s exact test *P* = 0.033 compared to WGT/WGD greater than 15 years before diagnosis).

A Poisson-like process model with a fixed rate of conversion from WGD to WGT per year (allowing for a fraction of WGD cases to never transform to WGT) fits the data well (Fig. 4g, red and green curves) and yields a relatively narrow estimate for the conversion rate of 11 ± 2% per year (Fig. 4g and Extended Data Fig. 5c–e). About 35% of tumors never reached triploidy even if the WGD event happened decades before diagnosis.

The relationship between FGA and the timing of the WGD/WGT event (Extended Data Fig. 5d) further supported an abrupt WGD-to-WGT transition. In a gradual process, cases with earlier WGD would slowly increase FGA over time. We instead found only a minor correlation in the years closest to diagnosis.

For other major somatic events, the earliest events in HPV^–^ cancers (+3q, +8q and *TP53*) occur around 30–40 years before diagnosis, while HPV^+^ early events (+3q and +9q) occur around 20–30 years before diagnosis (Fig. 4h). More recent clonal events, occurring less than 15–20 years before diagnosis, can be associated with clonal expansion, leading to primary disease. Although WGD events can occur very early, most occur ~20–25 years before diagnosis. All WGT tumors had the event 10 or more years before diagnosis. These time scales are consistent with the slow conversion rates from dysplasia to invasive carcinoma, on the order of 1 to 4% per year, reported for HPV^–^ HNSCC^53^.

Additionally, we estimated the timing of HPV integration events. We first aligned reads to the structural variation breakpoints at boundaries of 53 HPV integration sites across 11 individuals with WGS data (HPV can integrate multiple times per individual^39^) and analyzed multiplicities and CCFs (Extended Data Fig. 5f); 40 sites in 9 individuals showed good coverage. Many integration sites had high multiplicity on early gains, suggesting that integration of HPV is a pregain event contributing to initiating tumorigenesis. We also identified some lower-multiplicity and subclonal integration sites, suggesting that HPV integration is a continuous process that does not stop during progression (Fig. 4h and Extended Data Fig. 5f). Converting the timing of HPV^+^ integration into years before diagnosis suggests that initial HPV integration events can happen more than 25 years before diagnosis, with additional integrations throughout tumor development (Fig. 4h).

In summary, our timing analysis shows that (1) tumors are typically diagnosed many years after key driver events, which can happen at different ages (Extended Data Fig. 5), (2) founding events can occur 25–35 years or more before diagnosis, (3) WGT events do not seem to occur in the last ~10 years before diagnosis, and (4) HPV integration sites occur early in tumor development so that HPV, as expected, is the main contributor to HPV^+^ cancer initiation.

### Associations of heterogeneity and aneuploidy with outcome

Associations of HPV^–^ status and high MATH score with shorter overall survival in HNSCC are well established^49–52^. To see whether FGA and whole-genome events are similarly associated with outcome, we first examined a large subset of individuals whose survival is most strongly associated with intratumor heterogeneity: those without high-risk pathologic features who received either chemoradiation therapy or surgery without adjuvant therapy^51^. Initial analysis was restricted to individuals with HPV^–^ tumors to remove confounding associations. Among these 145 individuals (with 53 deaths), high FGA showed an association with overall survival similar to that of high MATH. High- versus low-score hazard ratios (HRs; with 95% confidence interval (CI_95%_)) were FGA (2.09; 1.05–4.2) and MATH (2.08; 1.11–3.9). Aneuploid tumors or tumors with whole-genome events leading to WGD or WGT had weaker associations with overall survival (aneuploid/diploid HR of 1.73 and CI_95%_ of 0.99–3.0; whole-genome event/none HR of 1.54 and CI_95%_ of 0.89–2.7; Fig. 5e–g).

To evaluate these associations in more individuals, including those with HPV^+^ tumors, while taking other outcome-associated variables into account, we extended our published survival model with MATH^51^ to incorporate the additional cases in this study. MATH was significantly associated overall with outcome (chi-square 17.75, 4 d.f., *P* = 0.0014, Wald test). Replacing MATH with FGA or WGD/WGT (Fig. 5e–g) in an otherwise identical model also showed significant associations with outcome (FGA: chi-square 12.68, 4 d.f., *P* = 0.013; WGD/WGT: chi-square 10.32, 4 d.f., *P* = 0.035). Aneuploidy class analyzed similarly was just above the significance threshold (chi-square 8.97, *P* = 0.06). Applying the Akaike information criterion (AIC), MATH (AIC of 1,350) had the strongest association with survival, although genomic instability measures had similar AIC scores (FGA, 1,355; WGD/WGT, 1,358; aneuploidy, 1,360).

Early genetic progression and aneuploidy development in tumors thus can be modeled computationally using WES data from primary tumors, providing findings about processes strongly associated with survival. Timing analysis is possible even when premalignant tissue is unobtainable, as in HPV^+^ HNSCC.

## Discussion

Our analysis of HPV^+^ HNSCC fills a major gap in knowledge about the progression of genetic events in this increasingly prevalent and clinically significant disease^35,37^, a cancer whose lack of premalignant tissue frustrated prior attempts at timing^39,40^. Analysis of HPV^–^ HNSCC verified that our computational approach could recapitulate the progression in classic HPV^–^ disease^8,54^ and extend the progression model to additional events in significant subsets of HPV^–^ disease. These results on cohorts of primary HNSCC specimens suggest that this approach could be applied to other types of cancer whose early genetic progression is unknown because premalignant tissue is difficult or impossible to obtain.

We extended the genetic progression model for HPV^–^ HNSCC to a total of 61 SNV or copy number alteration events, of which 17 had >40% prevalence. Our timing estimates agreed well with the Califano et al. model^8^ and with more recent studies that refined and extended it^30–34^. Comparing predicted mRT values (Fig. 1e) for events associated with the progression stages reported by Califano et al.^8^ (Fig. 1a) allowed us to map pathologic disease stages onto the mRT timeline. We determined when whole-genome events leading to WGD or WGT occur during HPV^–^ tumorigenesis, yielding estimates before or near the clinical development of invasive cancer.

Additionally, we identified genes with early mutations that likely play roles in HPV^–^ tumor initiation (*FBXW7*, *NOTCH1*, *CASP8*, *FAT1*, *NSD1*, *HRAS*, *EP300*, *CREBBP* and *KMT2D*), with over 60% of HPV^–^ HNSCCs having a mutation in at least one of these genes. Of those, we found anatomic and genetic differences among tumors, with *CASP8* mutations almost solely in the oral cavity and *NSD1* predominant in laryngeal tumors. By contrast, tumors with *NOTCH1* mutations, long suspected to play a role in HPV^–^ HNSCC, showed no anatomic preference or frequency or timing differences from other HPV^–^ HNSCC. Genes with mutations at later mRT (*PK3CA*, *NFE2L2*, *HLA-A*, *SPEN* and *KMT2C*) are more likely to be important for progressing to later stages of the disease.

In HPV^+^ HNSCC, gain of chromosome arm 3q and loss of arm 11q are both earlier and more frequent than in HPV^–^ HNSCC, indicating important early roles during HPV^+^ progression. We estimate that genomic integration of HPV viral DNA occurs early in tumor development, consistent with HPV being the main contributor to cancer initiation.

Arm 3q includes the *PIK3CA* locus, whose activation by copy number gain or mutation occurs often in HNSCC^43,55,56^; 74% of HPV^+^ and 62% of HPV^–^ HNSCC showed genetic alterations related to the *PIK3CA* locus. Gain of 3q was found at an earlier mRT in HPV^+^ tumors (0.05) than in HPV^–^ tumors (0.51; Figs. 1e and 2d,e), suggesting a particularly important early role for amplification of *PIK3CA* in HPV^+^ disease. Notably, however, mutations in *PIK3CA*, targeted by some therapies^57^, were relatively late in genetic progression in both classes of HNSCC. If tumors are less ‘addicted’^58^ to mutations that occur late in tumor development, therapies targeted against *PIK3CA* mutations might be of limited effectiveness.

With respect to loss of 11q in HPV^+^ HNSCC, *ATM* stands out as a candidate for early involvement in tumorigenesis. Although ATM improves the ability of episomal HPV to replicate^59,60^, its role in protection against double-stranded DNA breaks^60,61^ would be expected to inhibit genomic integration of HPV DNA. Losses of 11q also occur in HPV^–^ disease but much later at an mRT of 0.67 that we estimate to be near the development of carcinoma in situ (Figs. 1e and Fig. 2d,e).

Identifying genes whose loss along with chromosome arm 3p promote development of HPV^–^ HNSCC has long been an active area of interest^62^. The surprisingly early timing and high prevalence of 3p loss that we also found in HPV^+^ HNSCC suggests that studies of 3p genes will have clinical significance for all HNSCC.

Similarly surprising in HPV^+^ HNSCC is the prevalence of 17p loss, which includes the *TP53* locus, occurring in one-quarter of tumors (26 of 101). As HPV E6 leads to inactivation of the p53 protein product^42^, and *TP53* mutations occur in only 3% of HPV^+^ HNSCC, loss of additional genes on 17p presumably are important. Rather than the very early loss seen in HPV^–^ tumors, 17p loss occurs at intermediate stages of HPV^+^ disease progression (mRT of 0.66), suggesting that those genes are involved in later stages of tumor development.

Using a clock-like mutational signature to convert relative event timing estimates to years before diagnosis, we identified that early founding events can occur as many as 30–40 years before tumor diagnosis in HPV^–^ disease and 20–30 or more years in HPV^+^ tumors, with HPV integration also early in the development. WGD-to-WGT conversion seems to follow an abrupt transition model, with a specific transition probability per year.

This work helps clarify the nature of outcome-associated genomic disruption measures. Almost all tumors with a whole-genome event leading to WGT or WGD had high MATH values. Similar to high MATH, high FGA was also associated with shorter overall survival in HNSCC when therapies and standard clinical and pathologic characteristics were taken into account. Measures of genomic disruption can be used to classify individuals into high- and low-risk groups for monitoring disease with strategies such as minimal residual disease assays^63^. Identification of WGD timing before invasion might be used to identify HPV^–^ HNSCC at higher risk of progression. Other early drivers can be the basis of additional therapeutic trials, including in the preventive setting for high-risk individuals.

Applying our approach to other cancers whose premalignant tissue is seldom or never obtainable^14–22^, including multiple rare tumor types, could be of great clinical importance. Many such cancers have uncertain etiology, with effective treatment avenues less well defined than in more prevalent tumors. Establishment of progression trajectories, early drivers and similarity of progression to other cancer types can lead to development of improved disease models, better treatment strategies and clinical management and more accurate prediction of disease course.

## Methods

This research complied with all relevant ethical regulations. Human research on individuals at Massachusetts Eye and Ear (MEE) was approved by the MEE Human Studies Committee under protocol HSC 11-024H, with informed consent obtained from participants. Participants were not compensated.

### Participants, clinical data and tumors

We analyzed data from 486 individuals with head and neck cancer in TCGA^43^ and 45 individuals from MEE with oropharyngeal tumors whose WES data met quality control criteria (see below). TCGA clinical data were as in previous work^50,51^. Clinical data from MEE corresponding to TCGA data fields were extracted from participant records. Median time to death for 218 participants was 13.7 months (IQR of 8.4 to 26.5 months); follow-up time for the 313 last known to be alive was approximately twice as long (median of 29.4 months; IQR of 17.1 to 51.2 months). HPV status of TCGA tumors was assessed by RNA sequencing^43^; for MEE tumors, clinical HPV annotations were used. Clinical data are provided as Source Data for Fig. 5, including information on age and sex. Survival analysis was limited to individuals who survived more than 60 d after definitive pathologic diagnosis so that associations of outcome with adjuvant therapy could be ascertained.

TCGA tumor and control tissue or blood samples were as previously reported^43^. Tumor portions from MEE participants were frozen at liquid nitrogen temperature; participant-matched frozen blood provided control DNA for assessing tumor-specific mutations. Twenty-eight of the MEE tumor samples had two subsamples taken for separate processing.

### WES

Tumors from TCGA had undergone WES or WGS, as described^25^. Analysis proceeded from paired aligned bam files, which were inputted into a standard WES somatic variant-calling pipeline, including MuTect for calling somatic SNVs^64^, Strelka for calling small insertions and deletions^65^, deTiN for estimating tumor-in-normal contamination^66^, ContEst for estimating cross-participant contamination^67^, AllelicCapSeg for calling allelic copy number variants^68^ and ABSOLUTE for estimating tumor purity, ploidy, CCFs and absolute allelic copy number^68^. Artifactual variants were filtered out using a token panel of normals filter, a blat filter and an oxoG filter. For TCGA tumors in which the WES data did not yield sufficiently high-quality copy number data from AllelicCapSeg (361 tumors), we used HapSeg on single-nucleotide polymorphism (SNP) arrays as a substitute step. MEE tumors underwent a similar variant-calling pipeline as the TCGA tumors, but we did not substitute any AllelicCapSeg results with HapSeg on SNP arrays.

### Signature analysis for CoMut plots

We performed SBS signature analysis separately on the HPV^–^ and HPV^+^ subsets using SignatureAnalyzer^69,70^ on the somatic SNV calls resulting from the variant-calling pipeline.

### PhylogicNDT

We used PhylogicNDT to estimate relative event timing within individual tumors (SinglePatientTiming) and to combine timing information among tumors (LeagueModel). This analysis suite has been described in detail elsewhere^26^.

#### Within-tumor timing

After ABSOLUTE analysis^68^ to determine purity and ploidy, allele-specific SNV multiplicity estimates and purity-corrected copy number variation values from WES or WGS reads were used to set a within-tumor partial ordering of events. For example, on a chromosomal region that has been doubled, a tumor-specific mutation present at two copies is timed before the doubling, while a mutation present at only one copy is timed after the doubling. Tumor-specific genetic events are mapped on a *π* scale (as a probability distribution) from 0 to 1 (refs. ^23,26^), representing the first and last clonal genetic events. Estimated distributions are corrected for power to detect based on coverage profiles. The proportion of clonal events per megabase occurring before each genetic event in a tumor is defined as the *π* score for that event. Clonal events that cannot be timed are assigned a uniform *π* distribution, and the last clonal and all subclonal events are assigned *π* scores of 1, represented as a delta function *δ*(*π* − 1). Uncertainties arising from sequencing are incorporated into a distribution of *π* values for each event within the tumor. Thus within-tumor timing uses information from all tumor-specific SNV and copy number variation events.

#### Across-tumor timing

PhylogicNDT LeagueModel uses a Bayesian Monte Carlo Markov chain (MCMC) approach to combine single-tumor *π*-score distributions for each genetic event shared among a number of tumors into a consensus genetic relative timing progression. This consensus relative timing is generated by repeated sampling from the single-tumor *π* distributions of the shared events among multiple subsets of tumors. On this relative timing scale, a value of 0 represents the earliest shared event, and 1 is the last clonal shared event. The result is a distribution of relative timing scores for each shared event among the tumors based on comparison of within-tumor *π* scores among shared events. This was repeated for 200 iterations via resampling at an average of 63% without replacement, and the union of the relative timing score traces for each iteration was used as the final timing score distribution.

#### Timing comparisons

To compare consensus genetic progressions between HPV^+^ and HPV^–^ HNSCC or between subsets of HPV^–^ tumors, we performed a similar MCMC approach to the LeagueModel algorithm, but for each resampling iteration, we calculated the difference in the relative timing scores between subsets. Point estimates for timing differences were calculated as the median of the final trace, and one-way *P* values were calculated as the proportion of trace samples that was greater than 0. These were converted to two-way *P* values (*P*_2-way_ = 1 − 2|0.5 − *P*_1-way_|) and then to *q* values using multiple hypothesis correction.

### Real-time timing of somatic events

To estimate the real-time timing of somatic events, we used a robust linear regression model by removing the top and bottom 5% of slope outliers to fit the rate of clonal CpG>T (the ‘aging’ signature, SBS1) mutations (total number of mutations/number of covered CpG sites) corrected for copy number and multiplicity as a function of the age at diagnosis (Extended Data Fig. 5a) across different anatomical sites. The CpG>T rate per year and participant age were used to calculate the posterior distribution of the individual somatic event real-time timing measured from the *π*-score distribution calculated by using only CpG>T mutations with PhylogicNDT SinglePatientTiming. The resulting distribution for a specific event was then combined across participants to produce a cohort-level distribution for the typical time of the event in terms of years before diagnosis. Timing of HPV integration events was performed by aligning structural variation breakpoints and spanning reads at 53 integration sites across 11 individuals with available WGS data and performing a similar timing analysis as for mutations (using local copy number and clonal multiplicity). Because *APOBEC* mutations are often clustered near HPV integration sites, we excluded *APOBEC* mutations for the timing estimate. Often several integration sites were identified per participant, and sites on copy number regions with the earliest estimate of real time were used as representative for the participant.

### Measures of intratumor genetic heterogeneity

MATH was calculated per tumor sample as previously described^48^. For tumors represented by two separately sequenced samples, MATH values were averaged.

A tumor’s ploidy group was assigned based on the fraction of the genome in diploid, triploid and tetraploid states. Tumors with a diploid fraction of >0.65 were classified as diploid, non-diploid tumors with a fraction triploid of >0.35 were classified as triploid, and other non-diploid tumors were classified as tetraploid.

A whole-genome event was called if at least half of chromosome arms were amplified or at least four chromosome arms were amplified on both alleles. Further classification into triploidy (WGT) or doubling (WGD) was based on the corresponding ploidy group (triploid or tetraploid).

The FGA absent a whole-genome event was the fraction of the genome deviating by more than 0.2 from a value of one copy for either allele. With a whole-genome event, FGA was calculated as the fraction of genome deviating by more than 0.2 from a value of two copies for either allele.

### Statistical analysis and reproducibility

No statistical method was used to predetermine sample size. As there were no groups defined by experimental manipulations, no blinding was performed. Random resampling is inherent in PhylogicNDT, as described above.

Event prevalence comparisons, associations among measures of intratumor heterogeneity and other routine analyses were performed with standard statistical routines in Python or R. Frequentist tests (except for inherently one-sided chi-square statistics) were two sided. The Benjamini–Hochberg false discovery rate correction was applied to event prevalence comparison *P* values within each HNSCC subset. Survival analysis used the survival^71^ (version 3.3-1) and rms^72^ (version 6.3-0) packages under version 4.2.0 of R^73^, extending a previously described model for HNSCC survival^51^. Cox survival models were stratified by anatomic site to satisfy the proportional hazards assumption, and model calibration was checked by bootstrap resampling.

### Reporting summary

Further information on research design is available in the Nature Portfolio Reporting Summary linked to this article.

## Supplementary information

Reporting Summary

Supplementary Tables 1–7 Supplementary Table 1. Event prevalence comparison between HPV^+^ and HPV^–^ HNSCC. **a**, Number and percentage of cases having each of 66 genomic events for 421 HPV^–^ and 101 HPV^+^ HNSCCs. Fisher’s exact test two-sided *P* values are reported for comparison between HPV^–^ and HPV^+^ tumors. Benjamini–Hochberg false discovery rate-corrected *q* values are also shown. **b**, List of differentially prevalent events at *q* < 0.1. **c**, List of events not differentially prevalent (*q* < 0.1). **d**, Differentially prevalent events in **b** favored in HPV^–^ HNSCC. **e**, Differentially prevalent events in **b** favored in HPV^+^ HNSCC. Supplementary Table 2. Clonal event prevalence comparison between HPV^+^ and HPV^–^ HNSCC, like Supplementary Table 1 but restricted to clonal events. Supplementary Table 3. Event prevalence by anatomic subsite for HPV^–^ HNSCC. Breakdown by anatomic subsite of number and percentage of cases having specified genomic events: larynx, 114 tumors; oral cavity, 274 tumors; oropharynx, 33 tumors. Fisher’s exact test *P* values and Benjamini–Hochberg *q* values are as in Supplementary Table 1. Supplementary Table 4. Event prevalence comparison between *NSD1*-mutated and other HPV^–^ HNSCC, like Supplementary Table 1a–c but comparing 54 *NSD1*-mutated HPV^–^ HNSCC against 367 other HPV^–^ HNSCC. Supplementary Table 5. Event prevalence comparison between *CASP8*-mutated and other HPV^–^ HNSCC, like Supplementary Table 1a–c but comparing 50 *CASP8*-mutated HPV^–^ HNSCCs against 371 other HPV^–^ HNSCCs. Supplementary Table 6. Event prevalence comparison between *NOTCH1*-mutated and other HPV^–^ HNSCCs, like Supplementary Table 1a–c but comparing 70 *NOTCH1*-mutated HPV^–^ HNSCCs against 342 other HPV^–^ HNSCCs. Supplementary Table 7. Real-time timing estimates for whole-genome copy-number events.

## Data Availability

New WES data that support the findings of this study have been deposited in dbGaP under accession code phs003139.v1. Other human HNSCC genomic data were derived from the TCGA Research Network at http://cancergenome.nih.gov/. Source data are provided with this paper. All other data supporting the findings of this study are available from the corresponding authors on reasonable request.

## Source data

Source Data Source data for Figs. 1–5 and Extended Data Figs. 2 and 4–6.

## Code availability

The PhylogicNDT package is available for download from https://github.com/broadinstitute/PhylogicNDT.
