# Supplementary material for: Inferring early genetic progression in cancers with unobtainable premalignant disease
Source: Nat Cancer. 2023 Apr 20;4(4):550–63. doi: 10.1038/s43018-023-00533-y (PMC10132986; doi:10.1038/s43018-023-00533-y)
Supplement: Supplementary file 1 — Reporting Summary [file 43018_2023_533_MOESM1_ESM.pdf]

nature portfolio

Corresponding author(s): Gad Getz and James Rocco

Last updated by author(s): Oct 18, 2022

Reporting Summary

Nature Portfolio wishes to improve the reproducibility of the work that we publish. This form provides structure for consistency and transparency in reporting. For further information on Nature Portfolio policies, see our Editorial Policies and the Editorial Policy Checklist.

Statistics

For all statistical analyses, confirm that the following items are present in the figure legend, table legend, main text, or Methods section.

n/a	Confirmed	
☐	☒	The exact sample size (*n*) for each experimental group/condition, given as a discrete number and unit of measurement
☐	☒	A statement on whether measurements were taken from distinct samples or whether the same sample was measured repeatedly
☐	☒	The statistical test(s) used AND whether they are one- or two-sided<br>*Only common tests should be described solely by name; describe more complex techniques in the Methods section.*
☐	☒	A description of all covariates tested
☐	☒	A description of any assumptions or corrections, such as tests of normality and adjustment for multiple comparisons
☐	☒	A full description of the statistical parameters including central tendency (e.g. means) or other basic estimates (e.g. regression coefficient) AND variation (e.g. standard deviation) or associated estimates of uncertainty (e.g. confidence intervals)
☐	☒	For null hypothesis testing, the test statistic (e.g. *F*, *t*, *r*) with confidence intervals, effect sizes, degrees of freedom and *P* value noted<br>*Give P values as exact values whenever suitable.*
☐	☒	For Bayesian analysis, information on the choice of priors and Markov chain Monte Carlo settings
☒	☐	For hierarchical and complex designs, identification of the appropriate level for tests and full reporting of outcomes
☐	☒	Estimates of effect sizes (e.g. Cohen's *d*, Pearson's *r*), indicating how they were calculated

Our web collection on statistics for biologists contains articles on many of the points above.

Software and code

Policy information about availability of computer code

Data collection	No software was used for data collection, other than standard text and spreadsheet software for record keeping.
Data analysis	R (v 4.2.0) and its survival (v 3.3-1) and rms (v 6.3-0) packages; Python; PhylogicNDT (v1.0) available from https://github.com/broadinstitute/PhylogicNDT

For manuscripts utilizing custom algorithms or software that are central to the research but not yet described in published literature, software must be made available to editors and reviewers. We strongly encourage code deposition in a community repository (e.g. GitHub). See the Nature Portfolio guidelines for submitting code & software for further information.

Data

Policy information about availability of data

All manuscripts must include a data availability statement. This statement should provide the following information, where applicable:

- Accession codes, unique identifiers, or web links for publicly available datasets
- A description of any restrictions on data availability
- For clinical datasets or third party data, please ensure that the statement adheres to our policy

New whole exome sequencing data that support the findings of this study have been deposited in dbGaP under accession code phs003139.v1 and are available under standard repository policies. Other human head and neck squamous cell carcinoma genomic data were derived from the TCGA Research Network: http://

cancergenome.nih.gov/. Other data are provided as Source Data files. All other data supporting the findings of this study are available from the corresponding author on reasonable request.

Human research participants

Policy information about studies involving human research participants and Sex and Gender in Research.

Reporting on sex and gender	Sex was documented in TCGA or as provided by participants at Mass Eye and Ear (MEE; Boston MA USA) for their clinical records. 139 females, 392 males in total, consistent with prevalence in head and neck squamous cell carcinoma (HNSCC). After accounting for other covariates, sex was not associated with outcome. Thus the presentation does not distinguish by sex. Source data provide individual values.
Population characteristics	Patients diagnosed with primary squamous cell carcinoma of the head and neck (HNSCC), either under TCGA or at MEE. MEE recruitment from 2012 to 2014, with clinical data updated through 2019. Age range 19 to 90. Therapy received: Surgery only, 140; surgery plus adjuvant radiation, 130; chemoradiation as primary or adjuvant, 194. 6 participants who received primary radiation were omitted from survival analysis. Therapy received for 61 individuals could not be ascertained from TCGA data.
Recruitment	For MEE (non-TCGA) participants, staff requested participation from patients being evaluated for head and neck cancer in the clinic. As no change from standard-of-care therapy was involved, no selection bias resulting from a patient's choice to participate is expected.
Ethics oversight	MEE Human Studies Committee, under protocol HSC 11-024H

Note that full information on the approval of the study protocol must also be provided in the manuscript.

Field-specific reporting

Please select the one below that is the best fit for your research. If you are not sure, read the appropriate sections before making your selection.

☒ Life sciences ☐ Behavioural & social sciences ☐ Ecological, evolutionary & environmental sciences

For a reference copy of the document with all sections, see nature.com/documents/nr-reporting-summary-flat.pdf

Life sciences study design

All studies must disclose on these points even when the disclosure is negative.

Sample size	We started with the full TCGA HNSCC data set, which had already been found to provide reliable survival analysis but had low representation of HPV-positive tumors. We added further oropharyngeal tumor samples from MEE to provide a total of approximately 100 HPV-positive tumors, which we estimated would be enough to test this novel computational timing method on an important group of HNSCC. In practice, we found that a subset with as few as 50 samples could be analyzed for event timing. Data from 531 patients are presented.
Data exclusions	Patients whose exome sequencing of tumor samples did not pass quality control were excluded. For survival analysis, we restricted to those with follow up greater than 60 days to allow evaluation of the influence of adjuvant therapy on outcomes, as the association of genetic intratumor heterogeneity with outcome differs with therapy.
Replication	The PhylogicNDT timing methods involve randomized resampling of cases, providing estimates of distributions of timing that mimic repeated sampling from the underlying population. Calibration of Cox survival models was similarly evaluated by bootstrap resampling.
Randomization	As there were no experimental manipulations there was no need for randomization except for the case resampling noted above.
Blinding	With no groups distinguished by experimental manipulations, there was no need for blinding in data collection and analysis. Comparisons were made retrospectively between clinically or genomically defined groups of HNSCC, which required identification of group membership.

Reporting for specific materials, systems and methods

We require information from authors about some types of materials, experimental systems and methods used in many studies. Here, indicate whether each material, system or method listed is relevant to your study. If you are not sure if a list item applies to your research, read the appropriate section before selecting a response.

Materials & experimental systems

n/a	Involved in the study
☒	☐ Antibodies
☒	☐ Eukaryotic cell lines
☒	☐ Palaeontology and archaeology
☒	☐ Animals and other organisms
☒	☐ Clinical data
☒	☐ Dual use research of concern

Methods

n/a	Involved in the study
☒	☐ ChIP-seq
☒	☐ Flow cytometry
☒	☐ MRI-based neuroimaging
